# Influence of Cathode Channel Parameters and Fan Duty Ratio on Low Power Forced-Convection Open-Cathode Proton Exchange Membrane Fuel Cell Stack

**DOI:** 10.3390/mi14020286

**Published:** 2023-01-22

**Authors:** Jiaxu Zhou, Huichao Deng, Rui Xue, Yufeng Zhang

**Affiliations:** 1 School of Mechanical Engineering and Automation, Beihang University, Beijing 100191, China; 2 China Nanhu Academy of Electronics and Information Technology, Jiaxing 314001, China; 3 MEMS Center, Harbin Institute of Technology, Harbin 150001, China

**Keywords:** forced-convection OC-PEMFC stacks, Cathode channel parameter, fan duty ratio

## Abstract

The open-cathode forced-convection proton exchange membrane fuel cell has emerged as a viable option for portable energy sources. The forced-convection open-cathode mode, however, makes the cell’s performance sensitive to changes in the cathode channel and fan parameters. In this study, small fuel cell stacks with varying cathode channel depths, widths, and width–rib ratios were assembled, and the effects of different cathode channel parameters and fan duty ratios on cell performance were investigated. The experimental results show that changing the cathode channel parameters has a significant impact on oxidant supply. When the channel width is increased, the cell performance increases first, then decreases. The cell performance decreases as the channel width–rib ratio increases. The performance of the cell improves as the cathode channel depth increases. Furthermore, the experimental results show that decreasing the duty ratio of the fan and using moderate heating improves cell performance.

## 1. Introduction

The proton exchange membrane fuel cell (PEMFC) is a device that uses a chemical reaction to convert the chemical energy of hydrogen and oxygen directly into electricity. It is used in the automotive industry, and stationary and portable power generation fields due to its high efficiency, low operating temperature, and zero emissions [1,2,3]. Among many fuel cell systems, the open-cathode forced-convection proton exchange membrane fuel cell (OC-PEMFC) has received significant attention as a potential portable energy source for some small-scale power supply needs, such as an unmanned aerial vehicle, a small off-grid power supply, and so on, due to its simple structure and low parasitic power. In contrast to the closed-cathode PEMFC, the OC-PEMFC lacks complex air supply devices such as compressors and humidifiers, which reduce the fuel cell system’s volume and mass while increasing its energy density. OC-PEMFCs are classified into two types based on their air supply mode: self-breathing and forced-convection types [4]. Due to the natural convection of air into the cathode channel, the performance of self-breathing OC-PEMFC is inferior to that of closed-cathode cells. In forced-convection OC-PEMFC, the air is fed through a fan into the cathode which consumes negligible parasitic power when compared to a closed-cathode type. However, compared to the closed-cathode type, the cell’s performance is sensitive to cathode air supply conditions such as airflow and flow rate, which are heavily dependent on the cathode channel and fan parameters. Therefore, it is critical to investigate the effect of cathode channel and fan parameters on the performance of forced-convection OC-PEMFCs.

Several studies on the cathode channel parameter design of the OC-PEMFC have been conducted in recent years. Sasmito et al. [5] investigated and developed a mathematical model of an open-cathode PEMFC with an oxidant and cooling fan. According to the findings, there is a strong correlation between the height of the cathode flow field and stack performance. Sasmito et al. also use the Taguchi method to improve cell performance [6]. Kim et al. [7] investigated the effects of cathode channel size on cell performance and discovered that decreasing the cathode channel size improved cell performance at normal operating temperatures. At a low operating temperature and fan voltage, however, flooding limited the reduction in cathode channel size. Qiu et al. [8] proposed a three-dimensional air-cooled fuel cell model and examined the effect of cathode channel design. When the rib–channel ratio was set to 1.0, the channel width was reduced and the cell’s performance improved. Considering the influence of contact resistance when the rib–channel ratio was small, a rib–channel ratio within a reasonable range of around 3.0 was preferred for improved performance. Zhao et al. [9] investigated cathode channel design. When the bipolar plate thickness was 2 mm, the results indicated that the design channel size of 1.1 mm width, 1.3 mm depth, 1:0.7 width/landing, and 5° bending angle was the most optimal. Zhao et al. [10] investigated the effect of length–width ratio on cell performance. The findings indicate that the length–width ratio has a significant impact on cell performance. Furthermore, increasing the length–width ratio from 4.14 to 22.4 resulted in a 7% improvement in cell performance due to increased heat and mass transfer capabilities. Moreover, numerous novel cathode channel designs were tested. Thomas et al. [11] present a novel cathode channel design that provides uniform temperature and flow distribution. Baik et al. [12,13] developed a new separator in the rib region with a multi-hole structure, which improves cell performance at higher current densities. Lee et al. [14] developed a novel cathode channel to improve water retention under conditions of excess dry air supply. Wang et al. [15] investigated the effects of different cathode flow fields on cell operating conditions (parallel, pin-type, and metal foam). The result showed that the cell with the metal foam flow field outperformed the other flow fields in the baseline conditions. 

The influence of fans, in addition to cathode channel parameters, is a concern. Santarosa et al. [16] investigated the effect of fan voltage on cell performance. The experimental results showed that when the fan voltage was set to 5 V, the performance of the stack appeared to be optimal. Fan type (single fan or fans in series), fuel cell length, and separate air coolant channels were discovered by Sasmito et al. [5,17] to have a significant impact on the operating point and resulting stack performance. Pei et al. [18] investigated the effect of air flow rate and temperature on cell performance. The experimental results indicated that as the airflow rate increased, so did the cell’s performance. When the air flow rate exceeded 44.7 L/min or the temperature rose above 65°, performance decreased. Meyer et al. [19] used an electro-thermal map to investigate the effect of parasitic fan power on the net output power of a short fuel cell stack system. Ling et al. [20] investigated the effects of two cathode air supply modes (blow or draw air). The latter resulted in more uniform air velocities entering the stack and a 16% increase in cell performance. De las Heras et al. [21] conducted a comprehensive investigation of air-cooled proton exchange membrane fuel cells, paying special attention to the oxidant/cooling subsystem configuration. The experimental results indicated that the oxidant/cooling subsystem could condition the stack operation, and it was critical to control the stoichiometric rate values between the manufacturer’s required data to avoid stack degradation. Zeng et al. [22] discovered that the effect of fan speed on operating parameters varied depending on the load. Variable fan speed control must adapt to changes in fuel cell load and consider cell temperature, stack voltage, voltage uniformity, and parasitic power. Using a commercially available open-cathode PEM fuel cell system, Le et al. [23] investigated the effect of fan speed on cell performance and energy efficiency. The results indicated that the fan speed should be kept as low as possible to reduce auxiliary power consumption. Meanwhile, heating stack is effective to speed up the chemical reaction. Zhao et al. [24] investigated the air velocity distribution, polarization curve, single-cell voltage distribution, and temperature distribution of an open-cathode fuel cell stack equipped with a blower, blowing spoiler, and drawing air feed system. The results showed that using the spoiler improved stack performance by 7.3% by addressing the issue of air velocity in the middle of the stack. Drawing an air feed system improved heat dissipation capacity and temperature distribution uniformity, increasing cell performance by 7.9%. To optimise the distance between the fan and the stack, this could improve the full development of turbulence and the rate of heat transfer. 

There are many studies about the design of cathode channel parameters and fan selection in forced-convection OC-PEMFC. However, previous research on the design of cathode channel parameters has concentrated on simulation and single-cell experiments, and on fan selection has concentrated on single-cell and stack temperature, performance, and voltage. There have been few relevant experimental studies on the effect of cathode channel parameters on the stack and the effect of fan duty ratio on the stack electrochemical impedance spectroscopy (EIS). Compared with the single cell experiment and numerical simulation, the actual situation of the stack is more complicated. In this study, the performance characteristics of a small OC-PEMFC are investigated by varying the cathode channel parameters. Polarization curves, EIS, and surface temperature distribution were also investigated. Furthermore, the relationship between the fan duty ratio and stack performance, EIS, and surface temperature distribution is studied.

## 2. Experimental Section 

### 2.1. Experimental Setup

**Figure 1:**
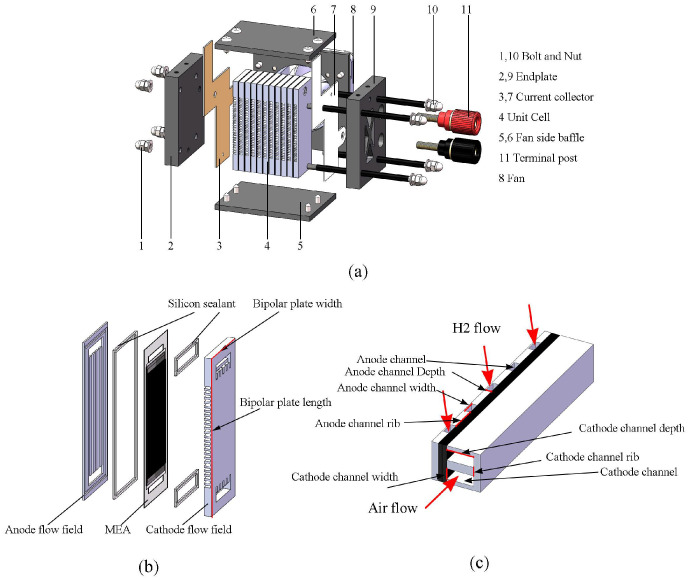
The 3D schematic of forced-convection OC-PEMFC: (**a**) fuel cell stack, (**b**) single cell, and (**c**) double channel model.

In this study, multiple 10-cell forced-convection OC-PEMFCs were designed and tested. A commercially coated catalyst membrane (M820.15, Gore, Newark, DE, USA) with an active area of 3.5 mm × 1.5 mm was used. The platinum loading was 0.1 and 0.3 mg/cm^2^ on the anode and cathode, respectively. GDL 280 and GDL 340 gas diffusion layers were used in the anode and cathode (CeTech Co., Ltd., Taiwan, China), respectively. To improve water retention, thicker carbon paper was used in the cathode. Computer numerical control technology was used to manufacture the self-designed graphite bipolar plate. The bipolar plate exhibited a thickness of 3 mm. Figure 1 depicts the stack design used in this study. As shown in Figure 1c, the anode and cathode channels are straight and perpendicular to each other. The width of the channel was set to 1 mm. The width of the channel, however, varied from 0.8 to 1.4 mm, and the width–rib ratio varied from 1:0.8 to 1:1.2. The processing parameters of the bipolar plate are shown in Table 1. The current collectors were gold-plated printed circuit boards. The end plate was developed from a 10 mm Bakelite plate. The sealing groove was sealed by filling it with liquid silicon sealant. The stack was secured with four M3 bolts. 

**Figure 2:**
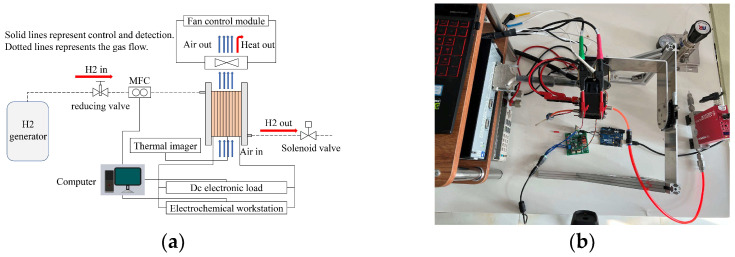
Experimental setup: (**a**) schematic diagram and (**b**) experiment platform.

The schematic diagram of the experiment is shown in Figure 2. The PEM fuel cell anode was fed dry 99.99% H_2_ from an H_2_ generator (LCH-500, LICHEN, Shanghai, China). A reducing valve was used to control the pressure of the hydrogen. To control and detect H_2_ flow, a mass flow controller was used (HORIBA Z714AGX, HORIBA, Kyoto, Japan). A 12 V fan controller was used to control an axial flow fan (RB0412H12B-6, Bomin Technology, Guangdong, China) by adjusting the Pulse width modulation (PWM) duty ratio to provide cooling and oxidant, maintain stack temperature, and avoid membrane dehydration. Data was recorded, and the hydrogen input was controlled using a computer. The polarization curve was tested with a DC electronic load (IT8812, ITECH Electronic Co., Ltd., Nanjing, Jiangsu, China). To achieve EIS, an electrochemical workstation (PARSTAT 3000 A, Princeton Applied Research, Berwyn, PA, USA) was used. A thermal imager was used to measure the surface temperature distribution (H21, Hikmicro Sensing Technology Co., Ltd., Hangzhou, Zhejiang, China).

### 2.2. Test Conditions

At the same operating temperature and humidity, the performance of a forced-convection OC-PEMFC was tested with different cathode channel parameters and fan duty ratios. All experiments were performed at ambient temperature and humidity levels. The ambient temperature was approximately 20–24 °C and the humidity was 40–50%. The anode H_2_ temperature was set to ambient, and the hydrogen pressure was set to 50 kpa. The hydrogen was supplied in a dead-end mode, with a solenoid valve for water removal opening every 16 s for 0.2 s. On the cathode, an axial flow fan was installed. The rated voltage and current of the fan were 12 V and 1.1 A, respectively. The fan duty ratio was varied from 80 to 40% to investigate the influence of fan cooling and air supply capacity on cell performance. Table 2 lists the specifics of various experimental schemes. 

For optimal performance, the membrane electrode assembly (MEA) must be activated prior to performance testing. During the active process, the load current ranged from 0.25 to 1 A with 0.25 A per point, and from 1 to 3 A with 0.5 A per point. Each load current was sustained for 3 min. The activation procedure was repeated until the performance was stable.

The galvanostatic mode was used to perform the EIS test, which measured ohmic and total polarization resistance. The EIS of the cell was tested at 0.76 A/cm^2^ current density (4 A load current). In the EIS test, the frequency ranged from 0.1 Hz to 20 kHz, and the amplitude of the AC was set to 5% of the load current. At 3, 4, and 5 A load currents, the surface temperature distribution was measured to evaluate cell performance. The EIS and surface temperature distribution test conditions were consistent with the cell performance test conditions.

## 3. Results and Discussion

### 3.1. Influence of Cathode Channel Width Variation

First, the cell performance of forced-convection OC-PEMFCs with cathode channel widths ranging from 0.8 to 1.4 mm and channel depth 2 mm was evaluated. The fan duty ratio was set to 80%, and the width–rib ratio was set to 1:0.8. Meanwhile, temperature distribution was tested at current densities of 3, 4, and 5 A. EIS was also tested at 0.76 A/cm^2^.

**Figure 3:**
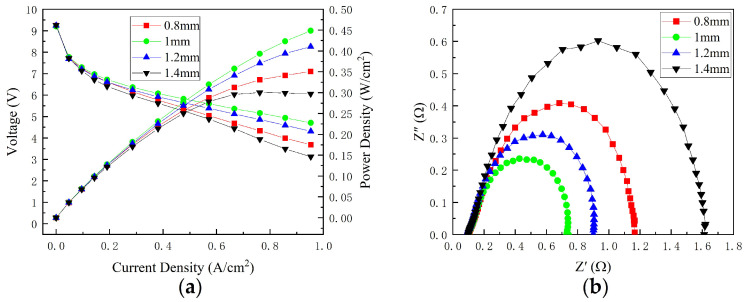
Performance of different channel widths: (**a**) polarization curves; (**b**) EIS at 0.76 A/cm^2^.

Figure 3a depicts polarization curves of various widths. It was discovered that the variation in cell performance with changing channel width was different at various current densities. The cell with a cathode channel of 1 mm produced exceptional results, and the cell with a channel width of 1.4 mm produced the worst results. When the current density was less than 0.19 A/cm^2^, the cathode channel width exhibited a negligible effect on cell performance. The cell performance was similar with cathode channel widths of 1 and 1.2 mm. When the current density was greater than 0.19 A/cm^2^, the cell’s performance first increased and then decreased as the cathode channel width increased. The cell with a channel width of 1 mm outperformed the cell with a channel width of 0.8 mm. The cell performance with a 0.8 mm channel width was lower than with a 1.2 mm channel width but higher than with a 1.4 mm cathode channel width. When the cathode channel width changed from 1 to 1.4 mm, the oxygen concentration in the membrane decreased due to a decrease in oxygen concentration in the rib region of the large-width channel. However, when airflow was relatively high, the oxygen concentration under the rib of a small-width channel could meet the reaction demand. The increase in the width of the channel from 0.8 to 1 mm reduced the pressure drop, improved oxygen mass transfer, and improved cell performance. Therefore, the effect of channel width on cell performance was complex.

Figure 3b depicts ohmic resistance and total polarization resistance at 0.76 A/cm^2^ for various cathode widths. The difference in ohmic resistance was only a few milliohms, and values were approximately 100 mΩ. This indicates that changing the cathode channel width has negligible effect on ohmic resistance at the same width–rib ratio. Ohmic resistance consists primarily of contact resistance, membrane resistance, gas diffusion layer resistance, bipolar plate resistance, and so on. Contact resistance is primarily determined by the contact area. The greater the contact area, the smaller the opening ratio. The opening ratios of channel widths of 0.8, 1, 1.2, and 1.4 mm were 54.9, 51.4, 54.9, and 56.0%, respectively. The contact resistance was the same. In this set of experiments, the MEA parameters remained constant. These findings also suggest that the membrane resistance is the same as the water content of the membrane. Furthermore, the total polarization resistance value first decreased and then increased with increasing channel width due to the effect of oxygen mass transfer, which is consistent with the variation law of cell performance with a changing channel width at 0.76 A/cm^2^.

**Figure 4:**
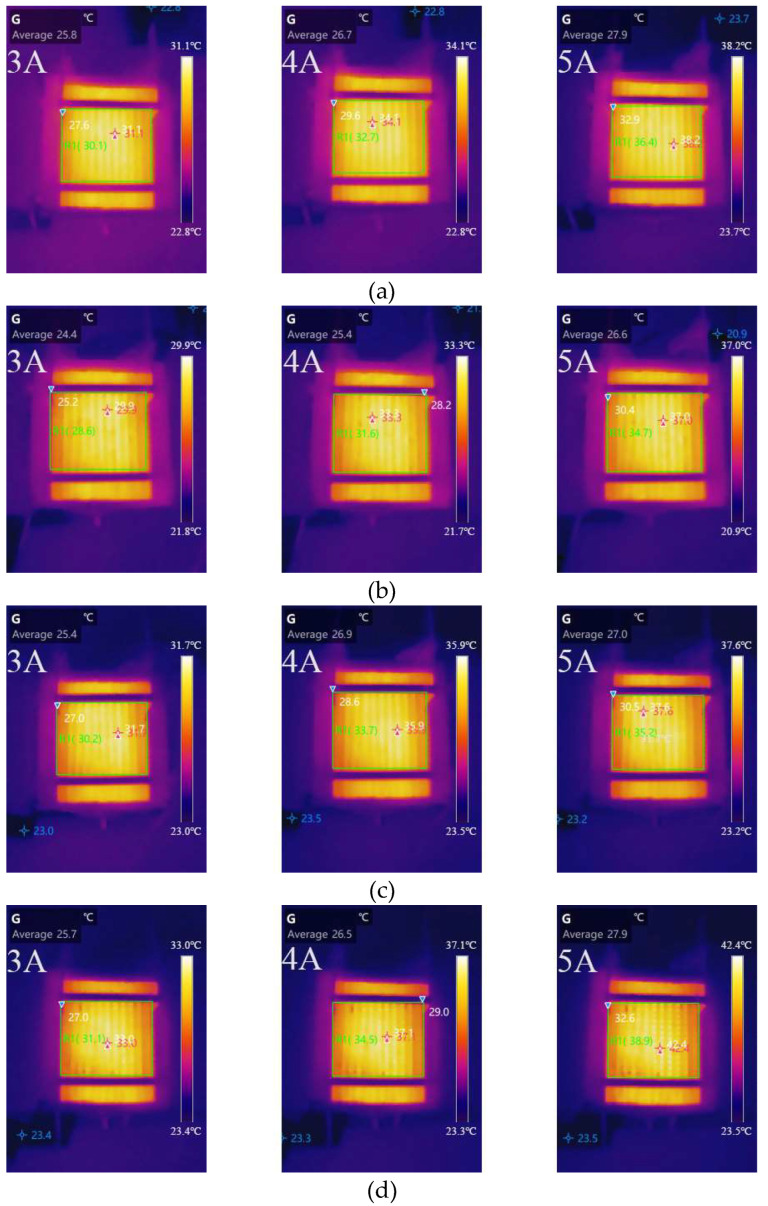
Temperature distribution of different channel widths at 3, 4, and 5 A: (**a**) 0.8 mm; (**b**) 1 mm; (**c**) 1.2 mm; (**d**) 1.4 mm.

Figure 4 shows the temperature distribution of cells of varying widths at 3, 4, and 5 A, respectively. Table 3 summarises the variation in the average temperature and the difference between the hottest and average temperature of different cathode channel widths with different currents. The temperature of the cell was determined by the cell’s waste heat and heat dissipation capacity. The average temperature increased with increasing load current at various cathode channel widths. The average temperature was the lowest in a cell with a channel width of 1 mm and the highest in a cell with a channel width of 1.4 mm. These results showed that the cell with a cathode channel width of 1 mm performed better in terms of heat dissipation. The size of the temperature difference can reflect the uniformity of the temperature distribution. When the current was 3 A, the temperature difference between cells of varying widths was less than 2 °C. As the load current and cathode channel width increased, so did the temperature difference. The greatest temperature difference was observed when the cell with a 1.4 mm cathode channel width was operated at 5 A. According to the findings, temperature distribution uniformity decreased as load current and cathode channel width increased. However, as the cathode channel width increased, the temperature difference variation decreased, and the difference in temperature distribution uniformity was small. Overall, the cell with a 1 mm cathode channel width was the most optimal of the cathode channel widths tested.

### 3.2. Influence of Cathode Channel Width–Rib Ratio Variation

When the channel depth was set to 2 mm and the channel width was set to 1 mm, the cell performance was tested with the cathode channel width–rib ratio changing from 1:0.8 to 1:1.2. The fan duty ratio was set to 80%. Meanwhile, temperature distribution and EIS were tested.

**Figure 5:**
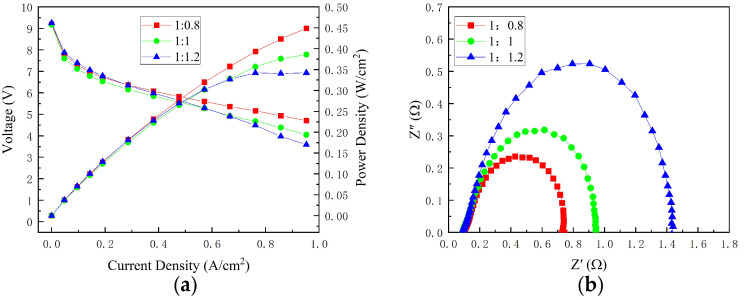
Performance of different channel width–rib ratios: (**a**) polarization curves; (**b**) EIS at 0.76 A/cm^2^.

The polarization curves of the cell with different width–rib ratios are shown in Figure 5a, and the effect of the cathode channel width–rib ratio variation on cell performance is revealed. At different current densities, the cell performance decreased when the width–rib ratio was changed from 1:0.8 to 1:1. The reason for this was that as the width–rib ratio increased, the oxygen concentration in the membrane decreased due to a decrease in oxygen concentration in the rib region. However, when the width–rib ratio was changed from 1:1 to 1:1.2, the performance variation law changed depending on the current density. When the current density was less than 0.57 A/cm^2^, the cell performance with a channel width–rib ratio of 1:1.2 was greater than that with a ratio of 1:1. This could be due to the relatively lower ohmic resistance of the cell with a 1:1.2 channel width–rib ratio when the oxygen supply was sufficient at a current density less than 0.57 A/cm^2^. When the current density exceeded 0.57 A/cm^2^, the cell’s performance with a channel width–rib ratio of 1:1.2 was worse than that of 1:1. When the current density was greater than 0.57 A/cm^2^, the oxygen concentration in the membrane decreased due to a decrease in oxygen concentration in the rib region and an increase in the width–rib ratio, which was the primary influencing factor for cell performance. When the current density exceeded 0.95 A/cm^2^, the cell’s performance with a channel width–rib ratio of 1:1.2 was significantly degraded due to non-uniform oxygen distribution.

Figure 5b shows the ohmic resistance and total polarization resistance at 0.76 A/cm^2^ for various cathode width–rib ratios. Different width–rib ratios exhibited the same ohmic resistance. According to the previous analysis, ohmic resistance was highly dependent on contact resistance variation. The changing trend of contact resistance was inversely proportional to the changing trend of the opening ratio. The opening ratios for the cells with width–rib ratios of 1:0.8, 1:1, and 1:1.2 were 51.4, 48.6, and 45.7%, respectively. When the contact area changed, the ohmic resistance value changed by only a few milliohms. The performance variation due to ohmic resistance variation was minimal. Otherwise, it can also be seen that the total polarization resistance increases with an increasing width–rib ratio, indicating a decrease in cathode oxygen mass transfer, which corresponds to a decrease in cell performance at 0.76 A/cm^2^.

**Figure 6:**
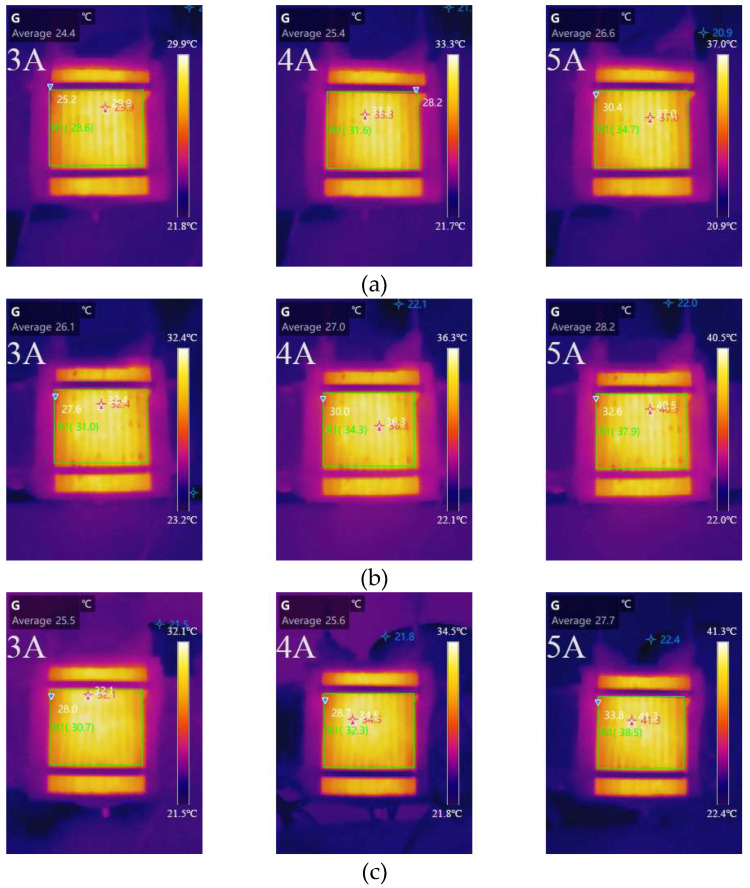
Temperature distribution of different width–rib ratios at 3, 4, and 5 A: (**a**) 1:0.8; (**b**) 1:1; (**c**) 1:1.2.

Figure 6 shows the surface temperature distribution of the different width–rib ratios of the cell at 3, 4, and 5 A, respectively. Table 4 depicts the variation in the average temperature and the difference between the hottest and average temperatures for various cathode channel widths and currents. The average temperature of the cell with a 1:1width–rib ratio was 1.4 °C when the load current was 3 A. The average temperature of the cell was the lowest with a 1:0.8 ratio. When the load current was 4 A, the average temperature of the cell was at its minimum and maximum, 1:0.8 and 1:1, respectively. However, at 5 A load current, the average temperature of cells with a 1:0.8 and 1:1.2 ratio was at a minimum and maximum, respectively. The cell with a cathode channel width–rib ratio of 1:0.8 performed better in terms of heat dissipation. When the current was 3 A, the temperature difference between different width–rib ratios was the same. The difference increased with the width–rib ratio when the current was in the range of 4 and 5 A. The temperature distribution was relatively uniform in the cell, with a cathode channel width–rib ratio of 1:0.8.

### 3.3. Influence of Cathode Channel Depth Variation

Then, forced-convection OC-PEMFCs with cathode channel depths of 1, 1.5, and 2 mm were tested for cell performance, EIS, and surface temperature distribution. The fan duty ratio was set to 80%, and the cathode channel width and the width–rib ratio of the cell were set to 1 mm and 1:0.8, respectively.

**Figure 7:**
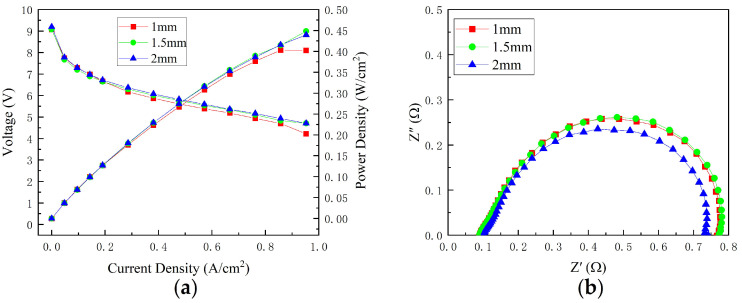
Performance of different channel depths: (**a**) polarization curves; (**b**) EIS at 0.76 A/cm^2^.

The variation in cell performance with cathode channel depth is shown in Figure 7a. When the current density was less than 0.19 A/cm^2^, the effect of cathode channel depth on cell performance was reduced because there was enough hydrogen and oxygen. When the current density was greater than 0.19 A/cm^2^, the cell’s performance gradually improved as the depth was increased. This could be attributed to a more uniform distribution of oxidants as the cathode pressure drop decreases with increasing cathode channel depth, causing the performance of all parts of the cell to be similar. The cell with 1 mm cathode channel depth performed the worst and exhibited a severe oxidant deficiency when compared to cells with 1.5 and 2 mm cathode channel depth. 

Figure 7b depicts the EIS at 0.76 A/cm^2^. It is possible to observe that the ohmic resistance values changed slightly as the cathode channel depth increased. Because only the depth of the cathode channel was changed, the contact resistance, bipolar plate resistance, and gas diffusion layer resistance remained unchanged. Furthermore, the total polarization resistance of the cell with a cathode channel depth of 2 mm was the lowest, followed by 1.5 and 1 mm, owing to the variation in distribution of oxidants as the cathode channel depth changed.

**Figure 8:**
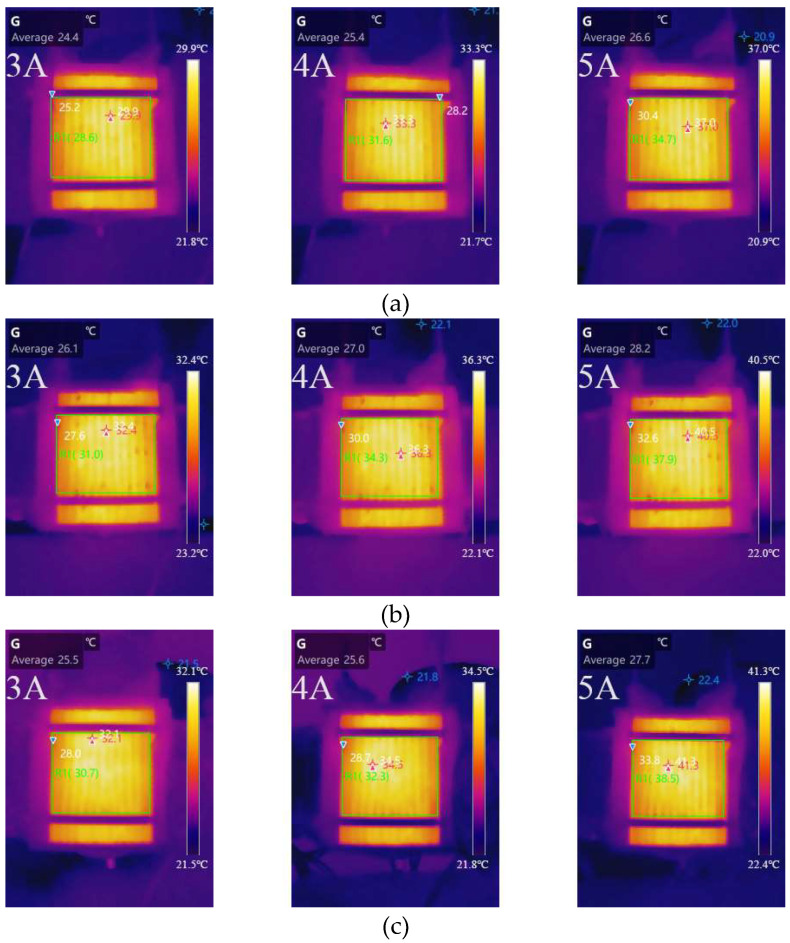
Temperature distribution of different channel depths at 3, 4, and 5 A: (**a**) 1 mm; (**b**) 1.5 mm; (**c**) 2 mm.

The surface temperature distribution of different cathode channel depths at 3, 4, and 5 A is shown in Figure 8. Table 5 shows the variation in the average temperature and the difference between the hottest and average temperatures of different cathode channel widths with different load currents. The temperature of the cell surface was discovered to decrease as the load current increased. At different load currents, the cell average temperature was the highest and lowest for cells with 1 and 2 mm cathode channel depths, respectively. The average temperatures were 28.6, 31.6, and 34.7 °C when the cell with a 2 mm cathode channel depth was operated at 3, 4, and 5 A load current, respectively. When the cell with a 1 mm cathode channel depth was operated at 3, 4, and 5 A, the average temperatures were 35.3, 39.6, and 44.3 °C, respectively. The cell average temperature difference between a 2 and 1 mm cathode channel depth was 6.7, 8.0, and 9.6 °C, respectively. The cell with a 1 mm cathode channel depth exhibited a lower heat dissipation potential. Furthermore, at different load currents, the temperature differences between the cells with 1.5 and 2 mm cathode channel depths were nearly identical. However, the temperature differences in the cell with a 1 mm cathode channel depth were greater than in the other cathode channel depth conditions. When the load current was 5 A, the temperature differences reached 3.4 °C. As the depth decreased, the temperature distribution became less uniform. When the temperature difference and average temperature under different depth conditions are compared, the cell with a 2 mm cathode channel depth performs better in terms of heat dissipation.

### 3.4. Influence of Fan Duty Ratio Variation

Furthermore, cells with fan duty ratios of 80, 60, and 40% were tested. The width of the cell cathode channel was 1 mm, the depth was 2 mm, and the width–rib ratio was 1:0.8. The EIS and the distribution of surface temperature were also tested.

**Figure 9:**
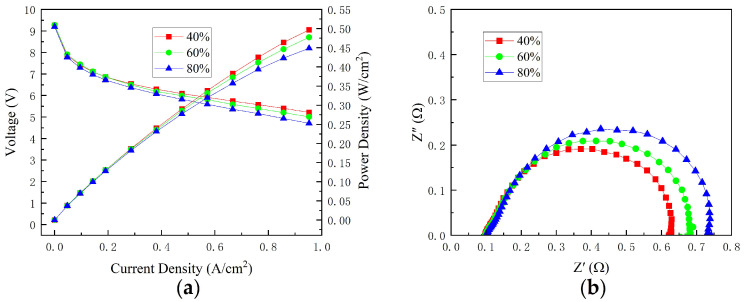
Performance of various fan duty ratios: (**a**) polarization curves; (**b**) EIS at 0.76 A/cm^2^.

Figure 9a depicts the performance curves of cells with varying fan duty ratios. When the current density was less than 0.19 A/cm^2^, the effect of the fan duty ratio on cell performance was minimal. When the current density was greater than 0.19 A/cm^2^, the cell’s performance decreased as the duty ratio increased. The cause of this performance change could be related to the cooling and air supply functions of the fan. Fans with duty ratio 40%, 60% and 80% can provide enough cooling to maintain the proper temperature and provide enough oxidant to complete the chemical reaction. However, fans with a low duty ratio have poor heat dissipation capabilities. The relatively high temperature accelerated chemical reactions and improved cell performance. In addition, with the increase in the fan duty ratio, membrane drying caused by a large flow rate may also be one of the factors causing the deterioration of stack performance. 

At 0.76 A/cm^2^, the ohmic and total polarization resistance are shown in Figure 9b. When the current density was 0.76 A/cm^2^, fans with different duty ratios could provide enough oxidant to complete the chemical reaction. It was observed that the ohmic resistance varied insignificantly. This shows that the loss of ohmic polarization caused by the change in airflow rate is not the main factor for the performance change. This may be because the designed stack has strong water generation capacity under high current conditions, and the change in air flow rate has little effect on the membrane water content. In addition, the total polarization resistance increased as the fan duty ratio increased, which is contrary to the fact that the oxidant supply decreased as the fan duty ratio decreased. The reason for this phenomenon may be that with the increase in duty ratio, the heat dissipation capacity is enhanced, the temperature of the cell decreases, the water flooding of the cell increases, the oxygen mass transfer is reduced, and the concentration polarization loss is increased.

**Figure 10:**
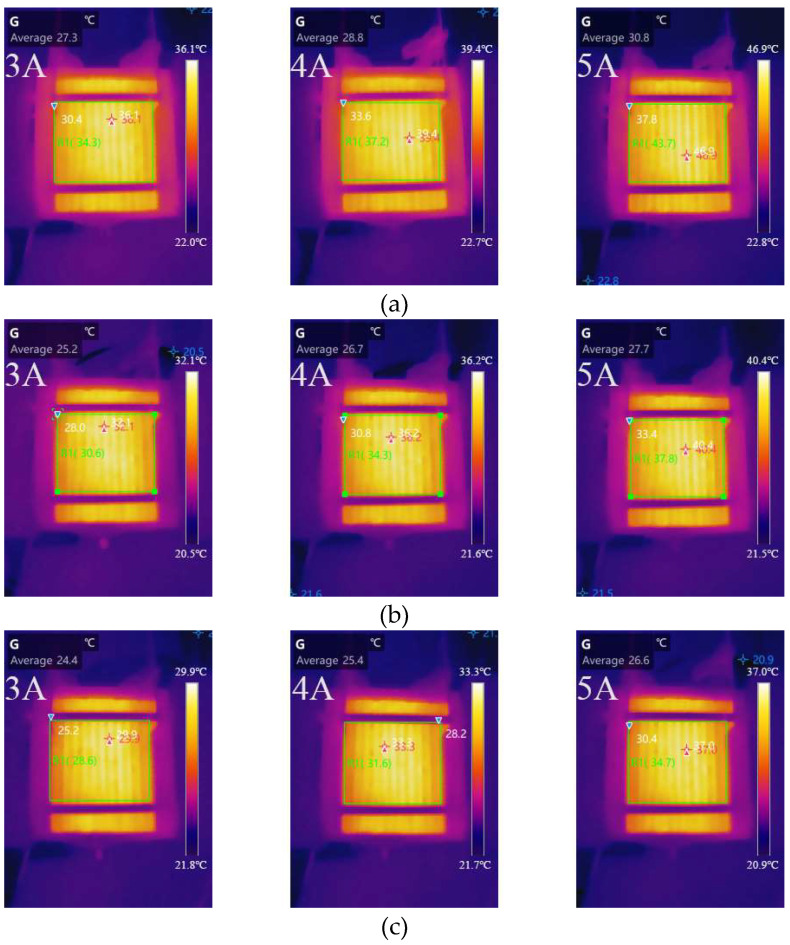
Temperature distribution of different fan duty ratios at 3, 4, and 5 A: (**a**) 40%; (**b**) 60%; (**c**) 80%.

Figure 10 depicts the surface temperature distribution of cells with different duty ratios at 3, 4, and 5 A. Table 6 depicts the variation in the average temperatures and the difference between the hottest and average temperatures of various cathode channel widths with varying load currents. The average temperature increased with the fan duty ratio decrease at different load currents, which was due to the fan heat dissipation capability decreasing with the fan duty ratio decrease. The average temperature reached 43.7 °C when the cell with a fan duty ratio of 40% was operated at a 5 A load current. Furthermore, at different load currents, the temperature difference increased as the fan duty ratio decreased. This appears to indicate that a high duty ratio can improve temperature distribution homogeneity, but it reduces the average temperature of the cell, which is unfavourable for the chemical reaction and water removal.

According to the cell performance curve and temperature distribution, the fan speed is inversely proportional to the cell performance when the oxidiser supply is satisfied. The smaller the duty ratio, the better the oxygen supply–demand balance and heat dissipation, and there is an appropriate temperature rise that is beneficial for cell performance. The temperature variation caused by the fan duty ratio is an important factor affecting the stack performance.

## 4. Conclusions

In this study, multiple forced-convection OC-PEMFC stacks with 10 cells were assembled and tested. Polarization curves, EIS, and surface temperature distribution were used to examine the effect of the cathode channel width, depth, width–rib ratio, and fan duty ratio on cell performance under ambient conditions. Therefore, the following conclusions can be drawn:

1. The effect of increasing cathode channel width on cell performance is complicated. When the width–rib ratio is 1:0.8, the influence of the changing cathode channel width on the cell varies depending on the load current. When the load current is less than 0.19 A/cm^2^, the cathode channel exhibits negligible effects on cell performance. When the load current exceeds 0.19 A/cm^2^, the influence of a pressure drop on oxidant supply and the influence of non-uniform oxidant concentration distribution caused by rib width changes take different precedence under different cathode channel widths. When the cathode channel width increases from 0.8 to 1 mm, the cell’s performance improves due to pressure drop reduction and oxygen mass transfer enhancement. When the cathode channel width increases from 1 to 1.4 mm, cell performance decreases due to the influence of non-uniform oxidant concentration distribution caused by rib width changes.
2. The influence of the cathode channel width–rib ratio increase on cell performance is negative when the current density is greater than 0.57 A/cm^2^ at a 1 mm cathode channel width, due to the increase in the trend of uneven distribution of oxidant concentration caused by the increase in the width–rib ratio. When the current density was less than 0.57 A/cm^2^, the cell performance with a width–rib ratio of 1:1.2 was better than the cell performance with a width–rib ratio of 1:1 because the cell with a width–rib ratio of 1:1.2 exhibited a lower ohmic resistance.
3. The increase in the cathode channel depth has a positive effect on cell performance. Because of the influence of the pressure drop on oxidant supply, cell performance gradually increases with increasing cathode channel depth.
4. The cell’s performance improves as the duty ratio of the fan decreases, owing to the increase in cell temperature increasing the chemical reaction rate.

The choice of the cathode channel parameters and fan duty ratio is crucial for stack design. Changes in these parameters have different effects on the stack under different operating conditions, as demonstrated by experimental analysis. According to the demand, the stack design should be reasonably adjusted. Future research directions include ensuring the output performance, reducing the quality, and maintaining a balance between output performance and output voltage, temperature, and load current.

## Figures and Tables

**Table 1:** The processing parameters of the bipolar plate.

Parameter	Value
Depth	Anode: 0.4 mm; Cathode: 1, 1.5, 2 mm
Width	Anode: 1 mm; Cathode: 0.8, 1, 1.2, 1.4 mm
Width–rib ratio	Anode: 1:2; Cathode: 1:0.8, 1:1, 1:1.2
Bipolar plate length	53 mm
Bipolar plate width	21 mm

**Table 2:** The details of different experimental schemes.

Case	Depth/Width/Width–Rib Ratio/Fan Duty Ratio	Annotations
1	1/1/1:0.8/80%	Different Depth
2	1.5/1/1:0.8/80%	
3	2/1/1:0.8/80%	
4	2/0.8/1:0.8/80%	Different Width
5	2/1.2/1:0.8/80%	
6	2/1.4/1:0.8/80%	
7	2/1/1:1/80%	Different Width–rib ratio
8	2/1/1:1.2/80%	
9	2/1/1:0.8/40%	Different Fan Duty Ratio
10	2/1/1:0.8/60%	

**Table 3:** Average temperature and temperature difference for different widths.

Variable	Current (A)	Width (mm)			
		0.8	1	1.2	1.4
Average temperature (°C)	3	30.1	28.6	30.2	31.1
	4	32.7	31.6	33.7	34.5
	5	36.4	34.7	35.2	38.9
Temperature difference (°C)	3	1	1.3	1.5	1.9
	4	1.4	1.7	2.2	2.6
	5	1.8	2.3	2.4	3.5

**Table 4:** Average temperature and temperature difference with different width–rib ratios.

Variable	Current (A)	Width–Rib Ratio		
		1:0.8	1:1	1:1.2
Average temperature (°C)	3	28.6	31.0	30.7
	4	31.6	34.3	32.3
	5	34.7	37.9	38.5
Temperature difference (°C)	3	1.3	1.4	1.4
	4	1.7	2.0	2.2
	5	2.3	2.6	2.8

**Table 5:** Average temperature and temperature difference at different depths.

Variable	Current (A)	Depth (mm)		
		1	1.5	2
Average temperature (°C)	3	35.3	32.1	28.6
	4	39.6	35.2	31.6
	5	44.3	38.8	34.7
Temperature difference (°C)	3	1.8	1.3	1.3
	4	2.2	1.8	1.7
	5	3.4	2.1	2.3

**Table 6:** Average temperature and temperature difference at different depths.

Variable	Current (A)	Fan Duty Ratio		
		40%	60%	80%
Average temperature (°C)	3	34.3	30.6	28.6
	4	37.2	34.3	31.6
	5	43.7	37.8	34.7
Temperature difference (°C)	3	1.8	1.5	1.3
	4	2.2	1.9	1.7
	5	3.2	2.6	2.3
